# Post-COVID-19 exacerbation of fibrodysplasia ossificans progressiva with multiple flare-ups and extensive heterotopic ossification in a 45-year-old female patient

**DOI:** 10.1007/s00296-021-04911-6

**Published:** 2021-06-10

**Authors:** Lovorka Grgurevic, Rudjer Novak, Stela Hrkac, Grgur Salai, Simeon Grazio

**Affiliations:** 1 Department of Anatomy, School of Medicine, University of Zagreb, Zagreb, Croatia; 2 Center for Translational and Clinical Research, Department of Proteomics, School of Medicine, University of Zagreb, Salata 11, Zagreb, Croatia; 3 Department of Rheumatology, Physical and Rehabilitation, Medicine University Hospital Center “Sestre Milosrdnice”, Zagreb, Croatia

**Keywords:** Myositis ossificans, COVID-19, Cytokines, Heterotopic ossification

## Abstract

Fibrodyplasia ossificans progressiva (FOP) is a rare hereditary disease, which has a variable course characterized by occasional flare-ups of heterotopic ossification (HO) in soft tissues that are followed by swelling, stiffness, pain and warmth. Here, we report for the first time a case of a 45-year-old female patient with known FOP recovering from COVID-19 with disease progression potentially linked with the viral illness. In December 2020 the patient contracted a mild form of COVID-19 infection without need for hospital admission. Since January 2021, the patient felt unwell, with occasional abdominal pain which progressively intensified. In March 2021 she presented with new onset of HO, complaining of pain, swelling and thickening sensation in the lower abdomen and left part of the neck. Computerized tomography (CT) and cytokine analysis were performed. CT scan revealed new heterotopic bone formation in multiple soft tissue areas of the neck indicating clear radiological progression. Radiotherapy, which has proven to be an efficient tool to control HO in this patient, was not able to halt HO formation after COVID-19 infection. Cytokine analysis of a plasma sample obtained during a flare-up after COVID-19 infection showed a significantly elevated pro-inflammatory cytokines compared to a flare-up panel prior to infection. Of the 23 analyzed levels of cytokines, a staggering number of 21 were above normal levels. This case is the first confirmation of uncontrolled post-COVID-19 effects in a FOP patient, which manifested with flare-ups followed by progressive HO, possibly caused by a thus far, never described form of post-COVID syndrome.

## Introduction

*Fibrodysplasia ossificans progressiva* (FOP) is a rare, but debilitating disease that affects 1:2,000,000 people worldwide. The disease causes bone formation in muscles and connective tissues, effectively entombing the patient in a “second skeleton”. The earliest skeletal malformations that are indicators of FOP can be present at birth, such as inward curving of the great toe. The disease progresses variably, and is characterized by episodes of exacerbation (flare-ups) with heterotopic ossification (HO) in soft tissues. HO appears spontaneously or after trauma to skeletal muscles and connective tissues and is characterized by swelling, stiffness, pain and warmth. Some lesions regress spontaneously, but most of them mature by an endochondral ossification pathway [1]. Although most cases are sporadic, the disease can be inherited through an autosomal dominant mutated ACVR1 gene, also known as Alk-2. This gene encodes an activin A receptor type I for bone morphogenetic proteins (BMP), which are members of the TGFβ superfamily [2]. Although animal studies have shown that Activin A inhibition could be highly effective in blocking progressive HO, this mutation alone is not sufficient for HO, since disease flare-ups are triggered by inflammation and activation of the innate immune system [3]. Studies have shown that innate immunity and cytokines are of great importance for HO occurrence and progression, furthermore, various cytokines have been implicated in HO pathogenesis [4, 5]. Previous research hypothesized that for the induction of HO in FOP patients an increase in at least one pro-inflammatory cytokine is both essential and sufficient to trigger the entire process of inflammatory cell influx [6]. Coronavirus disease (COVID-19) is caused by SARS-CoV-2 and represents a global public health concern [7]. It can present with various symptoms and degrees of severity, some of which can be life-endangering and even fatal [8].

In this report, we describe the case of a 45-year-old female patient with FOP who suffered from a mild form of COVID-19 infection after which she experienced intense FOP exacerbation (i.e., flare-up). This case report is complemented by patient’s plasma cytokine profile analysis and literature review on the association of FOP and HO with COVID-19. To the best of our knowledge, this is the first report of a FOP patient recovering from COVID-19 and the first time COVID-19 convalescence has been linked to FOP progression.

## Case presentation

**Fig. 1:**
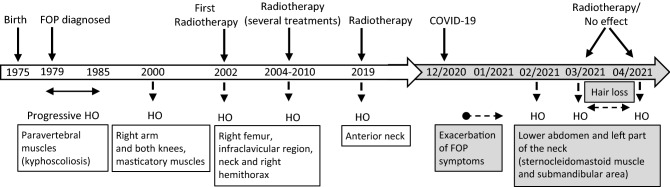
Progression timeline in Patient 1 with FOP. Disease progression is depicted chronologically according to timepoints of HO occurrence and their respective location, as well as timepoints of radiotherapy. Events and disease exacerbation after COVID-19 infection are highlighted in grey

A 45-year-old female patient with FOP presented to the Rheumatology Outpatient Clinic in March 2021, complaining of pain and swelling of the lower abdomen and left part of the neck. The patient was diagnosed with FOP at the age of four when a red, painful gristle appeared on her back, which later turned into bone (Fig. 1). This was followed by progressive ossification of the paravertebral muscles, leading to kyphoscoliosis, making her unable to erect herself by the age of ten. During her teenage years, occasional use of etidronate and methylprednisolone seemed to somewhat slow down formation of new HO foci. However, at the age of 25 (year 2000), new HO occurred in her right arm and both knees, followed by development of eating difficulties due to the affection of masticatory muscles (Fig. 1). The patient herself reported subjective improvement of HO shortly after diagnostic X-ray, which led to the decision to start radiotherapy treatment. At the age of 27, she received a dose of 10 Gy (delivered in five fractions) on her right arm and 8 Gy (delivered in two fractions) on both knees. This halted HO progression over the following 2 years, as confirmed by X-ray. Keeping with the progressive nature of FOP, in 2004 new HO foci started developing. Radiation therapy was again attempted several times until 2010 on her right femur, infraclavicular region, neck and right hemithorax, where she was treated with 2 Gy in two, 6 Gy in six, 4 Gy in four, and 3 Gy in three fractions. The patient positively responded to the therapy and was well over the period of 9 years. However, another flare-up event occurred in 2019, with swelling and HO in the anterior part of the neck. A cytokine analysis was performed along with routine laboratory processing and radiotherapy treatment (4 Gy in 4 fractions), which resulted in immediate alleviation of bone formation and good function (Fig. 1).

**Fig. 2:**
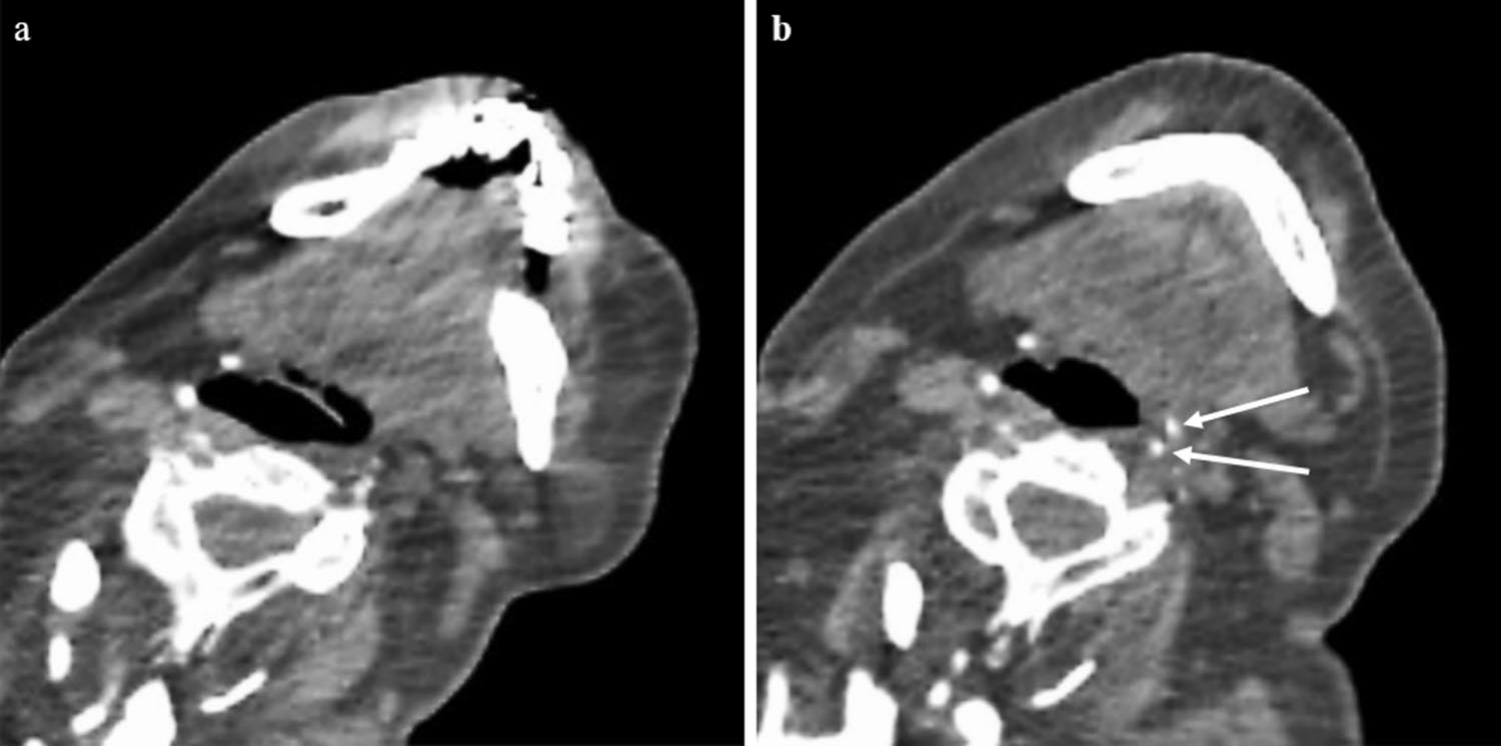
Axial computed tomography (CT) images of the FOP patient taken pre- (**a**) and post-COVID-19 infection (**b**). New foci of heterotopic ossification surounding the oropharynx at the level of cervical vertebrae No 5 (C5) appeared after COVID-19 infection convalescence (white arrows). Due to disease progression resulting in severe ambulatory restriction, movement restraint and fixed mandibula, there is a difference in neck position compared to baseline scan (CT machine*: Toshiba Aquilion Large Bore 16 slices CT scanner, Toshiba Medical Systems Corporation, Tokyo, Japan*)

In December 2020 the patient and her household family members contracted the COVID-19 infection, as confirmed by RT-PCR test on a sample obtained from a nasopharyngeal (NP) swab for SARS-CoV-2. Main symptoms were mild fever (max 37.5 °C), mild cough, fatigue and sweating. She was treated as an outpatient with azithromycin and paracetamol and over the next 2 weeks, she recovered completely without the need for hospital admission and with negative control NP swab test for SARS-CoV-2. FOP exacerbation had restarted approximately 4 weeks post-COVID-19 convalescence and has thus continued steadily (Fig. 1). Since January 2021, the patient did not feel well, which was accompanied by changes in blood pressure and fatigue, with occasional abdominal pain attributed to a possible urinary tract infection. In February 2021, abdominal pain progressively intensified after she was examined by an abdominal surgeon on several occasions for suspected acute abdomen. A month later, she presented with new onset of HO, complaining of pain, swelling and thickening of tissues in the lower abdomen and left part of the neck (sternocleidomastoid muscle). Upon admission, a clear progression of HO was observed on CT scan of the same neck area from September 2020 (Fig. 2). Radiotherapy treatment was used again on the lower abdomen (4 Gy in four fractions) and on the left part of the patient’s neck (4 Gy in two fractions). Unfortunately, the treatment was not successful as forewarned by the patient’s residual feeling of uninterrupted swelling. In April 2021, the patient reported a new area of progression in the left submandibular area, marginal to previous radiotherapy field. A CT scan confirmed new HO located in the oropharynx, indicating clear radiological progression (Fig. 2). She again received 4 Gy in 2 fractions to the affected area, however, with no apparent clinical response. Neck movement is currently completely restricted, as well as movement of other parts of the patient’s body. Unfortunately, if such FOP progression persists, she is expected to develop inability to swallow and be respiratory compromised.

Radiotherapy was proven as an efficient tool to control HO in this patient. In addition to radiotherapy, during periods of flare-ups and HO, the patient was treated with corticosteroids (methylprednisolone) up to doses of 64 mg per day with rapid decrease in dosage. She also continuously takes several anti-inflammatory and analgesic medications: a selective COX-2 inhibitor, a combination of paracetamol and opioid analgesic, a leukotriene and a histamine receptor antagonist. However, after SARS-CoV-2 infection, FOP has progressed, and radiotherapy has been unable to halt HO formation. Pathophysiological mechanisms to explain these observations are lacking. No damage or dysfunction of other vital organs was found in the patient after COVID-19 infection. Among the usual laboratory blood tests performed before and after COVID-19 infection, the only difference observed was in the values of C-reactive protein (CRP). Slightly elevated levels of CRP were observed in April 2021 (8.7 mg/L; reference values < 5 mg/L) in comparison to September 2020 and March 2021, which were within the reference interval (5.1 mg/mL). Increased hair loss was observed 3 months after infection as part of post-COVID-19 syndrome; no other signs or symptoms were observed or reported by the patient.

**Fig. 3:**
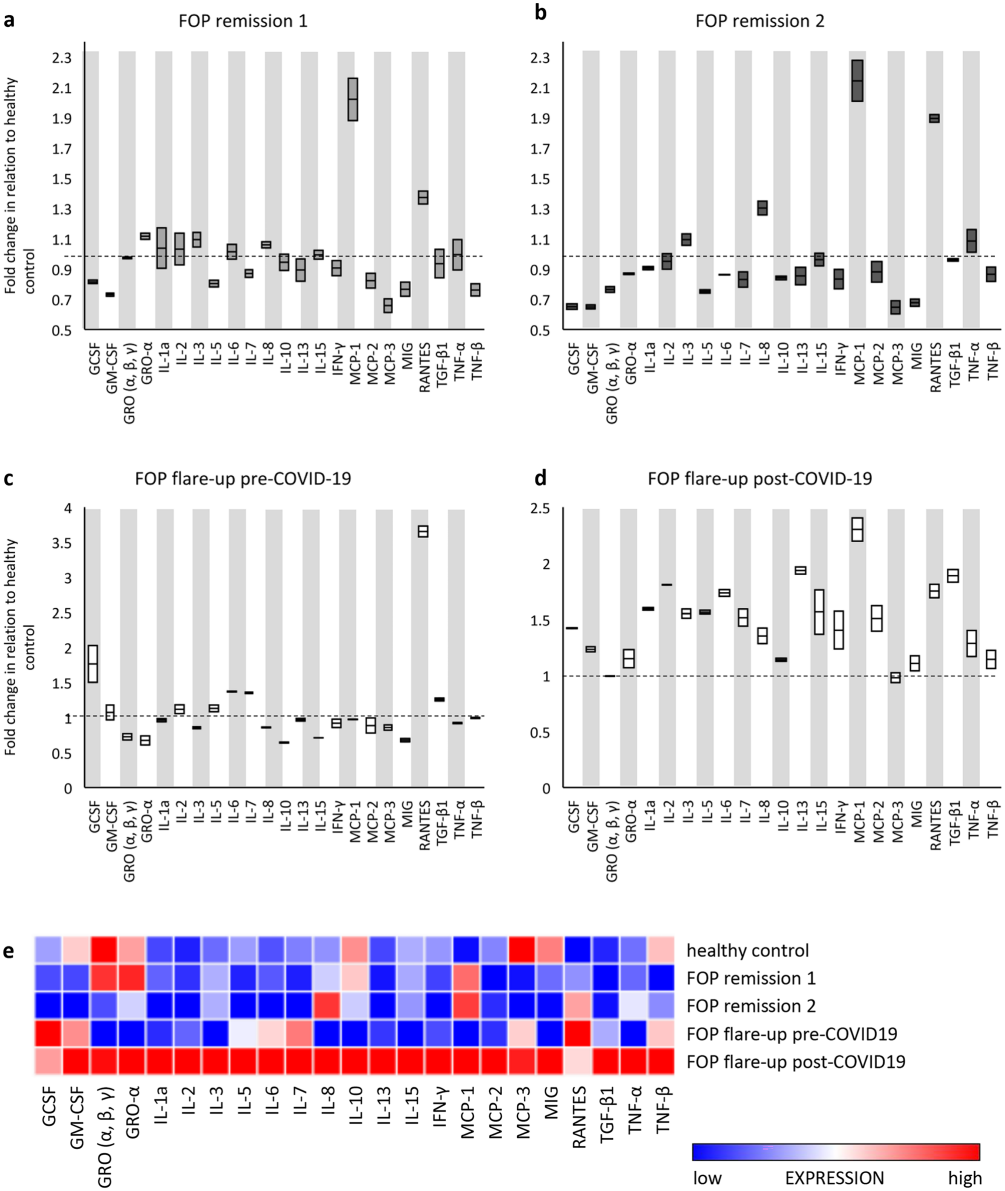
Cytokine profiles of FOP patients presented as relative fold change in relation to healthy control: **a** FOP patient 2 (control) during a disease remission phase; **b** FOP patient 3 (control) during a disease remission phase; **c** FOP patient 1 (presented in the case) during a disease flare-up (sample obtained prior to COVID-19 infection); **d** FOP patient 1 (presented in the case) during a disease flare-up (sample obtained 3 months after COVID-19 infection); **e** Heat map depicting cytokine expression profiles of *healthy controls* and FOP patients during remission (*FOP remission 2 and 3*, in comparison to *FOP pre- and post-COVID-19 flare-ups* in patient 1

Being aware of the rarity of her disease, this patient frequently contributes towards understanding of FOP by providing plasma samples for scientific purposes which are obtained for the purposes of evaluation and treatment during flare-ups (namely, she only consents her blood to be drawn when it is absolutely indicated for fear that the microtrauma of venipuncture might trigger HO). A panel of 23 pro- and anti-inflammatory mediators was used to screen her plasma before and after COVID-19 infection. The results were compared to three control samples: two female FOP patients in remission that were not COVID-19 positive and a plasma pool of ten healthy women of the same age group with no bone disorders. This study was approved by the institutional ethics committee and all participants signed an informed consent form. Blood samples were drawn into standard vacuette citrate tubes with special care as not to induce HO by needle insertion. Participants’ plasma samples were screened for differences in cytokine levels using the human cytokine antibody array (#ab133996, Abcam) and analyzed using the Chemi Doc imaging system (BioRad) according to manufacturer's recommendations. Quantification of chemiluminescent signals was performed with ImageJ 1.52a software (NIH) [9]. The cytokine expression levels are presented as fold change relative to the expression levels of the healthy-pool control sample (Fig. 3). Plasma cytokine expression during a flare-up in the period after COVID-19 infection, showed strikingly higher levels of most of the analyzed cytokines, as compared to the pre-COVID-19 panel of the same patient (Fig. 3c–e).

### Search strategy and literature overview

We searched the PubMed and Scopus databases for original articles, reviews, letters, short communications and notes in English from 1971 for PubMed and from 2004 for Scopus to the latest date, which was May 2021 for both. Our search strategy included looking for titles, abstracts, and keywords, using the following terms: (“Fibrodysplasia ossificans progressiva” OR “FOP”) AND (“COVID-19” OR “SARS-CoV-2”), including a search of PubMed by the aforementioned MeSH terms [10]. The search was further expanded by replacing either of the terms: “COVID-19” with “viral illness” or “FOP” with “HO” (heterotopic ossification), in all combinations. The references in each selected study were reviewed to identify other relevant articles. The search results are shown in Table 1.

**Table 1:** Relevant articles pertaining to FOP/HO and COVID-19/viral illness

Reference	Article type	*N*^a^	Sex	Age	Viral illness	Underlying FOP^d^	HO^e^ site
Scarlett et al. [11]	Survey	123	53 Mb 70 F^c^	3–72	Influenza-like	Yes	Neck, back, trunk, groin, legs
Meyer et al. [12]	Case-series	4	4 M	39–74	COVID-19	No	Shoulders
Aziz et al. [13]	Case-series	2	1 F	43, 51	COVID-19	No	Hip, shoulder

^a^*N* number of participants

^b^*M* male

^c^*F* female

^d^*FOP* fibrodysplasia ossificans progressive

^e^*HO* heterotopic ossification

## Results

Our search for FOP and COVID-19-related biomedical literature, conducted as previously described, did not yield any results. Upon furthering our search strategy, three articles were found (Table 1): two that relate HO to COVID-19 and another that connects FOP flare-ups with a viral illness (Influenza type B).

## Discussion

FOP is recognized as one of the most debilitating diseases known to medicine. In addition to its severe and often unpredictable clinical course, there is, despite extensive research, no specific curative or preventive treatment. The exact mechanism and triggers for HO in FOP remain largely unclear. It is recognized that the ACVR1 mutation leads to enhanced Smad1/5/8 signaling and altered responsiveness to canonical (BMP) and non-canonical (Activin A) ligands [14]. However, ACVR1 mutation and Activin A are not solely responsible (or sufficient) for HO to occur. Previous research shows that involvement of immune cells, such as macrophages and mast cells, as well as various cytokines are of great importance in the process of HO [5]. In a previous study of plasma cytokine levels in FOP patients, it was hypothesized that RANTES (Regulated on activation, normal T-cell expressed and secreted/CCL5) might be a potential trigger and/or indicator of HO [6]. Cytokine analysis of a sample obtained during a flare-up prior to COVID-19 infection from the same patient, showed a significantly lower amount of elevated pro-inflammatory cytokines compared to the post-COVID-19 panel. These laboratory findings were also reflected in better pre-COVID-19 reaction to radiotherapy, which was followed by symptom alleviation. In our patient’s post-COVID-19 cytokine panel, most notable elevations were found in MCP-1, RANTES and IL-13, which are cytokines with emerging roles in bone remodeling and cortical bone formation [15–17]. We also found a significant elevation of plasma IL-5, which has been reported to cause ectopic bone formation in animal models [18]. Of the 23 analyzed cytokines, a staggering number of 21 were above normal (healthy control) expression levels. Among them, IL-1, IL-6, IL-8, Interferon-γ, TNFα and GM-CSF were prominently increased. Limitations of the cytokine analysis we conducted include the lack of remission phase sample of our patient, inability to precisely quantify cytokine concentrations and possibility that crucial cytokines for understanding of the observed HO are not part of the cytokine panel kit we employed. In spite of these limitations, our findings suggest an upward trend from our patient’s “baseline” flare-up values. We suggest that such cytokine hyperproduction is the result of an imbalance in natural regulation mechanisms that are used to eliminate pathogens and avoid an exaggerated immune response. In this case, an overabundance of cytokines probably initiated marked worsening of the underlying disease. We observed no other systemic manifestations or damage to vital organs that are usually present in conditions with similar cytokine disturbances [19]. We hypothesize that COVID-19 might have acted as a long-term trigger for disease exacerbation, i.e., as an inductor of flare-ups which progressed to sites of HO. The effects of influenza-like illness on FOP patients have been documented before, as patients reported flare-ups which occurred after influenza-like symptoms [11]. Interestingly, in patients of the aforementioned study, the neck and trunk were commonly affected sites—which were also affected in our FOP patient after COVID-19 infection. However, these reported flare-ups occurred within days of viral illness onset, whereas our patient experienced disease worsening 4 weeks after COVID-19 infection convalescence with no improvement to this moment. This might be due to the specific nature of this disease, namely, its associated post-COVID-19 syndrome, which occurs beyond 4 weeks from the onset of symptoms. Tissue damage and innate immune response with inflammatory cytokine production may contribute to the various sequelae of this syndrome [20]. We argue that tissue damage caused by post-COVID-19 syndrome might be the potential trigger of our patient’s notable disease progression, as well as the associated elevation of inflammatory cytokines. Notably, several instances of non-FOP-related HO after severe forms of COVID-19 have been reported, which further supports our hypothesis [12, 13].

## Conclusion

This case is the first case report of uncontrolled post-COVID-19 effects in a FOP patient, which manifested with consecutive disease flare-ups followed by progressive HO. The episode was possibly caused by a, thus far, never described form of post-COVID syndrome, which regularly affects the most sensitive and already disturbed parts of the body (*locus minoris resistentiae*). Prolonged ectopic ossification after COVID-19 infection, with marked changes in the cytokine profile of our patient, may point towards potential mechanisms contributing to the pathophysiology of post-acute COVID-19 syndrome, which in this case might be accompanied by aberrations of the innate and adaptive immune system. Understanding of these mechanisms is limited and further study is needed to improve FOP patient care during the COVID-19 pandemic and beyond.

## Data Availability

The data sets used and/or analyzed during the current study are available from the corresponding author on reasonable request.
